# Survival and prognostic factors in conventional central chondrosarcoma

**DOI:** 10.1186/s12885-018-4741-7

**Published:** 2018-08-24

**Authors:** Julian Fromm, Alexander Klein, Andrea Baur-Melnyk, Thomas Knösel, Lars Lindner, Christof Birkenmaier, Falk Roeder, Volkmar Jansson, Hans Roland Dürr

**Affiliations:** 1 Musculoskeletal Oncology, Department of Orthopedics, Physical Medicine and Rehabilitation, University Hospital, Ludwig-Maximilians-University, Campus Grosshadern, Marchioninistr. 15, D-81377 Munich, Germany; 2 Department of Radiology, University Hospital, LMU Munich, Munich, Germany; 3 Institute of Pathology, University Hospital, LMU Munich, Munich, Germany; 4 Department of Internal Medicine III (Oncology), University Hospital, LMU Munich, Munich, Germany; 5 Department of Radiotherapy and Radiation Oncology, University Hospital, LMU Munich, Munich, Germany; 6 CCU Molecular Radiation Oncology, German Cancer Research Center (DKFZ), Heidelberg, Germany

**Keywords:** Chondrosarcoma, Surgery, Margin status, Recurrence, Prognostic factors

## Abstract

**Background:**

Chondrosarcoma is the second most frequent primary malignant bone tumor. Treatment is mainly based on surgery. In general, wide resection is advocated at least in G2 and G3 tumors. But which margins should be achieved? Does localization as for example in the pelvis have a higher impact on survival than surgical margins themselves?

**Methods:**

From 1982 to 2014, 87 consecutive patients were treated by resection. The margin was defined as R0 (wide resection), R1 (marginal resection) or, R2 if the tumor was left intentionally. All patients were followed for evidence of local recurrence or distant metastasis. Overall and recurrence-free survival were calculated, significance analysis was performed.

**Results:**

In 54 (62%) cases a R0 resection, in 31 (36%) a R1 and in 2 (2%) patients a R2-resection was achieved. Histology proved to be G1 in 37 patients (43%), G2 in 41 (47%) and G3 in 9 cases (10%). 5-year local recurrence-free survival (LRFS) was 75%. Local recurrence-free survival showed a significant association with the margin status and the localization of the tumor with pelvic lesions doing worst. Metastatic disease was initially seen in 4 patients (4.6%), 19 others developed metastatic disease during follow-up. Overall survival of the entire group at 5 and 10 years were 79 and 75%, respectively. The quality of surgical margins and the presence of local recurrence did not influence overall survival in a multivariate analysis. Pelvic lesions had a worse prognosis as did higher grades of the tumor, metastatic disease and age.

**Conclusions:**

The mainstay of therapy in Chondrosarcoma remains surgery. Risk factors as grading, metastatic disease, age and location significantly influence overall survival. Margin status (R0 vs. R1) did influence local recurrence-free survival but not overall survival. Chondrosarcomas of the pelvis have a higher risk of local recurrence and should be treated more aggressively.

## Background

Following Osteosarcoma, chondrosarcoma (CS) is the second most frequent primary malignant bone tumor accounting for approximately 20% of all bone sarcomas [1]. It constitutes a heterogeneous group of tumors characterized by the production of cartilaginous matrix [2]. Central (conventional) CS represents about 75% of the group. With the introduction of the current WHO classification in 2013 Chondrosarcoma grade I (now officially termed atypical cartilaginous tumor) was reclassified as an intermediate (locally aggressive) tumor, better reflecting its clinical behavior [2]. In these difficult cases, the differential diagnosis towards benign enchondromas is based on a combination of pathology, radiology and clinical features and hence requires a close multidisciplinary assessment [3].

Treatment is mainly based on surgery and chemotherapy is less effective because of a low mitotic index and poor vascularity [4, 5]. Radiotherapy is effective but requires substantial dosage [6].

In general, wide resection is advocated at least in G2 and G3 tumors. It is assumed that patients with CS have an excellent prognosis after adequate surgery [4] but reviewing the literature and our own results, such assumptions should be looked at in a more detailed fashion. Even the G1 lesions have a risk of metastasis of 6% [7]. There is no clear consensus on what exactly constitutes “adequate surgery”. Which margins should be achieved? Does localization as for example in the pelvis have a higher impact on survival than surgical margins taken for themselves? In a metaanalysis on 1114 patients published in 2015, the surgical margin were not identified as an independent predictor of overall survival [8]. In consequence, the traditional dogma of adequate margins, as stated by some authors [9, 10] had to be called into question.

The main aim of this retrospective study was to analyze a homogenous group of patients with primary central CS of bone, treated at a single tumor center. We sought to determine prognostic factors for overall and local recurrence-free survival. Secondary aim was to asses our own results on the background of the published data.

## Methods

From 1982 to 2014, 87 consecutive patients with chondrosarcoma of the extremities, pelvis and trunk wall were treated at our institution. All tumors had a diagnosis of chondrosarcoma based on histological features and immunohistochemistry.

Prior to surgical resection, predominantly magnetic resonance imaging (MRI) and in some cases computed tomography (CT) was used to define size and localization of the tumor. A CT scan of the chest was performed to determine the presence or absence of metastatic disease.

All patients underwent surgical resection. The margin was defined as R0 if a rim of healthy tissue around the lesion was present (wide resection) or R1 if the margins were contaminated but the tumor capsule remained closed (marginal resection). In select patients, a planned partial resection was performed in order to avoid severely mutilating surgery. This was classified as a R2 resection.

### Statistical analysis

All patients were followed for evidence of local recurrence (LR) or distant metastasis in general by regional MRI scans and chest radiographs. Clinical outcomes of local recurrence (LR), local recurrence-free survival (LRFS) and overall survival (OS) were used for assessment. LRFS and OS were defined either as the time from surgery to the first occurrence of local recurrence or to death from any cause. For statistical analysis, overall and local recurrence-free survival were calculated according to the Kaplan-Meier method. Significance analysis was performed using the Log-Rank, the Chi-Square test or the Cox proportional-hazards regression model. A *P* value of less than 0.05 was considered statistically significant. The data analysis software used was MedCalc® (MedCalc Software, Ostend, Belgium).

## Results

The median age of the 54 male and 33 female patients was 51.7 years (mean 50.3, range 15–83). The lower extremity was involved in 44 cases (29 femur, 18 of them proximal; 11 tibia, 10 of them proximal; fibula and feet 2 each), the upper extremity in 10 (7 humerus, 5 of them proximal; radius, ulna and hand 1 each), the pelvis in 21 and the trunk in 12 (8 scapula, 2 ribs, clavicle and thoracic spine 1 each) patients. Fifty patients (57.5%) showed extraosseous tumor growth.

The median duration of symptoms prior to diagnosis was 9 months (range, 0–358) and the majority of patients (44 = 52%) complained of pain, 11 (13%) of swelling. A pathologic fracture led to the diagnosis in 6 (7%) patients. Neurological impairment or restriction of movement was seen occasionally. 70 (81%) patients had a biopsy or histology before surgery. In 2 cases, the biopsy was interpreted as a cartilaginous exostosis and in 3 cases as an enchondroma. Four patients had already undergone surgery at other institutions by means of intramedullary nailing or by resection and endoprosthetic reconstruction. In these cases, the tumor had either gone unidentified or it had been underestimated. Only 4 patients had metastatic disease initially.

Resections of the tumor alone were performed in 42 cases (48%), resections and reconstructions with megaendoprostheses in 24 cases (28%), amputations in 11 patients (13%) and curettages in 10 instances (11%). A wide (R0) resection was performed in 54 (62%) cases, a marginal (R1) resection in 31 cases (36%) and an R2-resection in 2 (2%) patients. With pelvic lesions, 48% of surgical margins were either R1 or R2, at the lower extremity 41%, at the upper extremity 20% and at the trunk 25% (n.s.). Histology proved to be G1 in 37 patients (43%), G2 in 41 (47%) and G3 in 9 cases (10%).

In 20 patients (23%), surgical revisions due to complications had to be performed. This included:

Nine revisions due to dislocation or loosening of implants or bone grafts, 6 deep infections, 2 hematomas, and more aggressive tumor resection, neurological impairment and vessel injury in 1 case each.

In 63 surviving patients, the median follow-up time from surgery to last information on the patient was 68 months (range, 0–379). One patient was lost to follow-up less than 12 months after surgery, 8 patients had a follow-up of 12–24 months. Twenty-four patients deceased during follow-up.

**Fig. 1:**
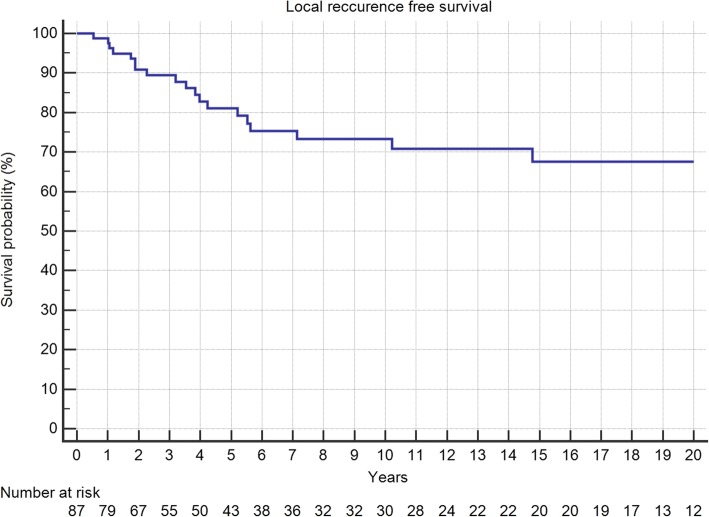
Local recurrence-free survival in 87 patients with central chondrosarcoma

**Fig. 2:**
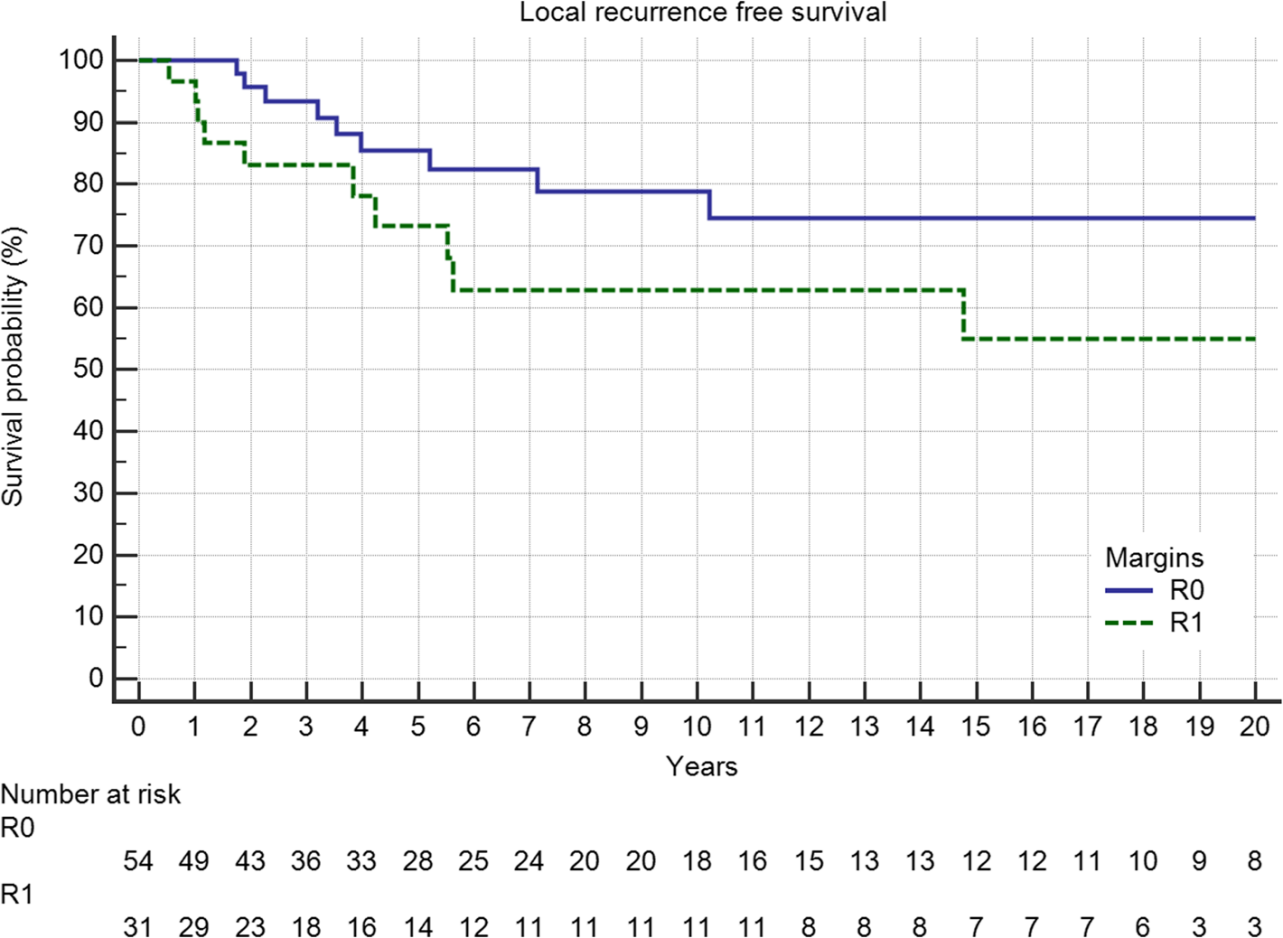
The impact of surgical margins on local recurrence-free survival in R0 and R1 resected patients (*p* = 0.05)

**Fig. 3:**
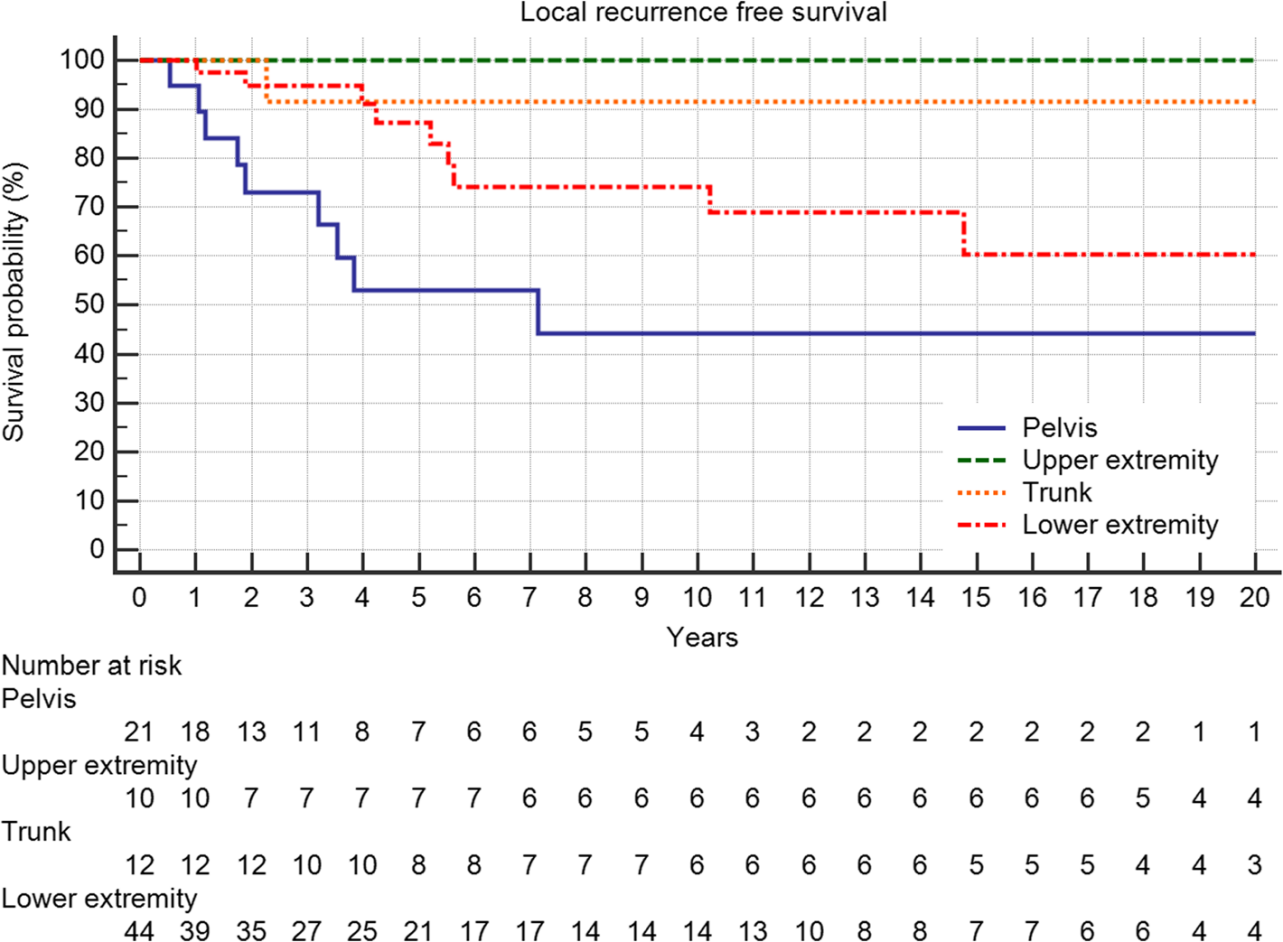
The impact of tumor localization on local recurrence-free survival (*p* = 0.0532)

Five-year local recurrence-free survival was 75%. In total, 21 (24%) patients developed local recurrences, of which 52% occured in the first 12 months and 81% in the first 24 months after surgery (Fig. 1). The latest LR was seen after 10 years. Local recurrence-free survival showed a significant association with the margin status and the localization of the tumor with pelvic lesions doing worst (Table 1, Figs. 2 and 3). In multivariate analysis, both kept significance.

**Table 1:** Factors influencing local recurrence (margin status, location) and local recurrence free survival

Local recurrence	No	Yes	*p*-value	5-year LRFS	10-year LRFS	*p*-value
R0	45 (83%)	9 (17%)	0.1025^*^	84.7%	81.4%	0.0204^+^
R1	20 (65%)	11 (35%)		61.9%	61.9%	
R2	1 (50%)	1 (50%)		0%	0%	
Upper Extremity	10 (100%)	0 (0%)	0.0568^*^	100%	100%	0.053^+^
Lower Exremity	35 (80%)	9 (20%)		79.0%	79.0%	
Pelvis	12 (57%)	9 (43%)		55.7%	44.7%	
Trunk	9 (75%)	3 (25%)		75.0%	75.0%	

^*^Chi-squared test;

^+^Logrank test

Metastatic disease was initially seen in 4 patients (4.6%). One of those patients stayed free of disease after resection, 19 others developed metastatic disease during follow-up. At final follow-up, 22 (23%) patients had metastatic disease, 13 of which were located in the lung, 3 in the spine, one in the femur, one in visceral organs and 4 in multiple localizations. Only 5 of these patients were alive with disease at final follow-up. Of these 22 patients with metastatic disease, only 8 also had a LR (36%) whereas 20% of non-metastasized patients had LR which was not statistically significant. Grading showed a trend towards metastatic disease in follow-up with 14% in G1, 30% in G2 and 44% in G3 tumors but without statistical significance (*p* = 0.0815).

**Fig. 4:**
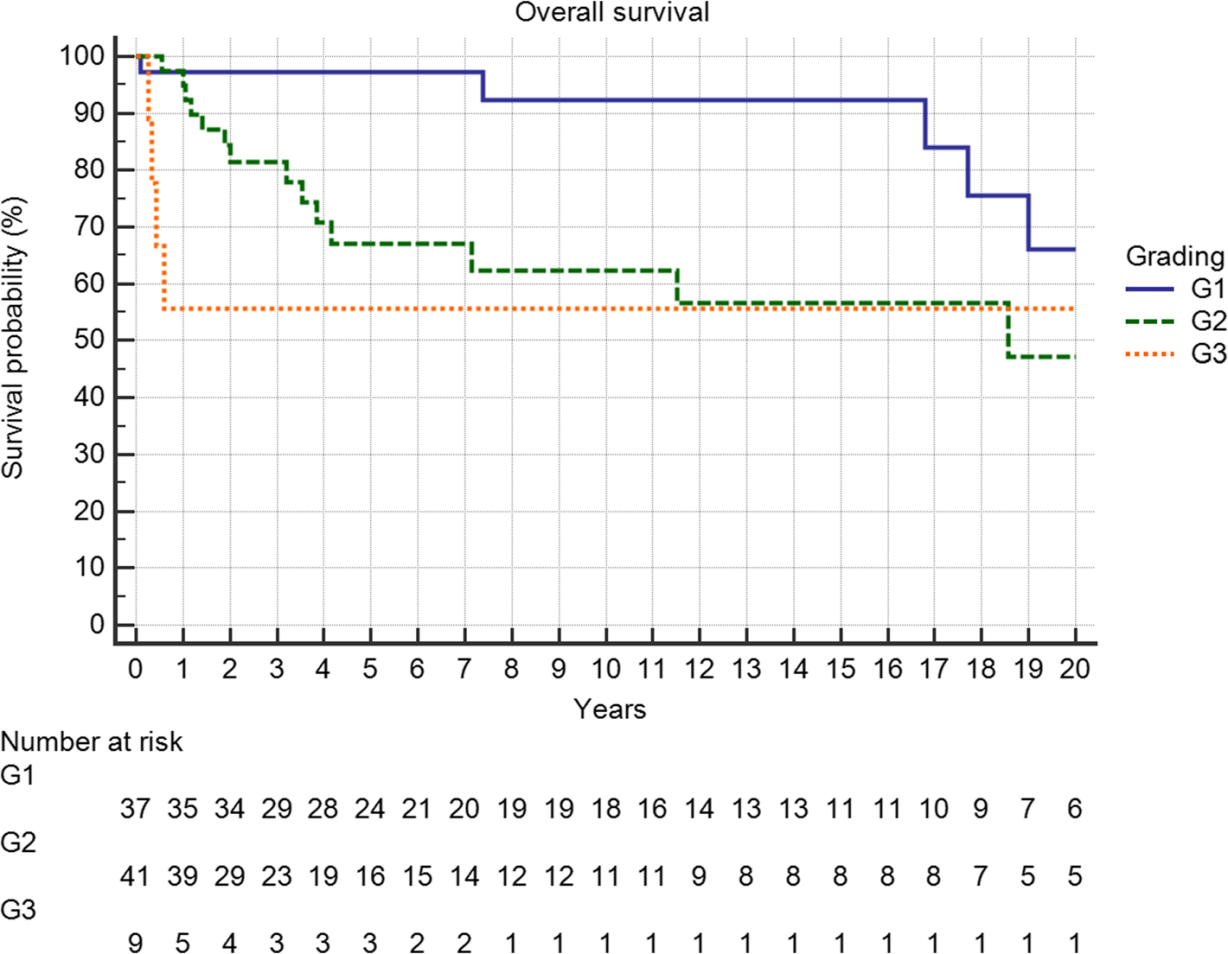
Overall survival is strongly influenced by tumor grading (*p* = 0.0099)

**Fig. 5:**
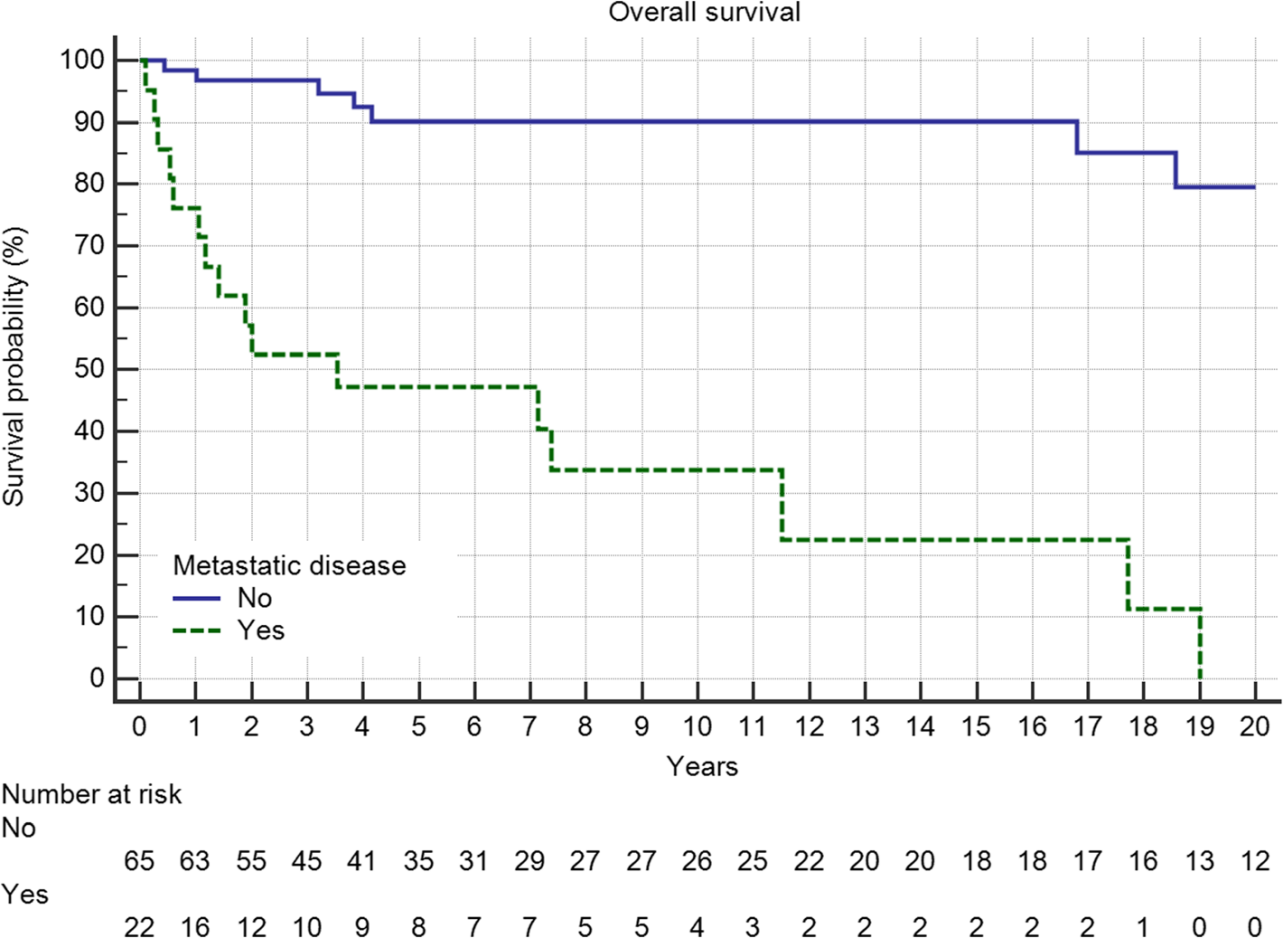
Metastatic disease in 22 patients significantly deteriorates overall survival (*p* < 0.0001)

**Fig. 6:**
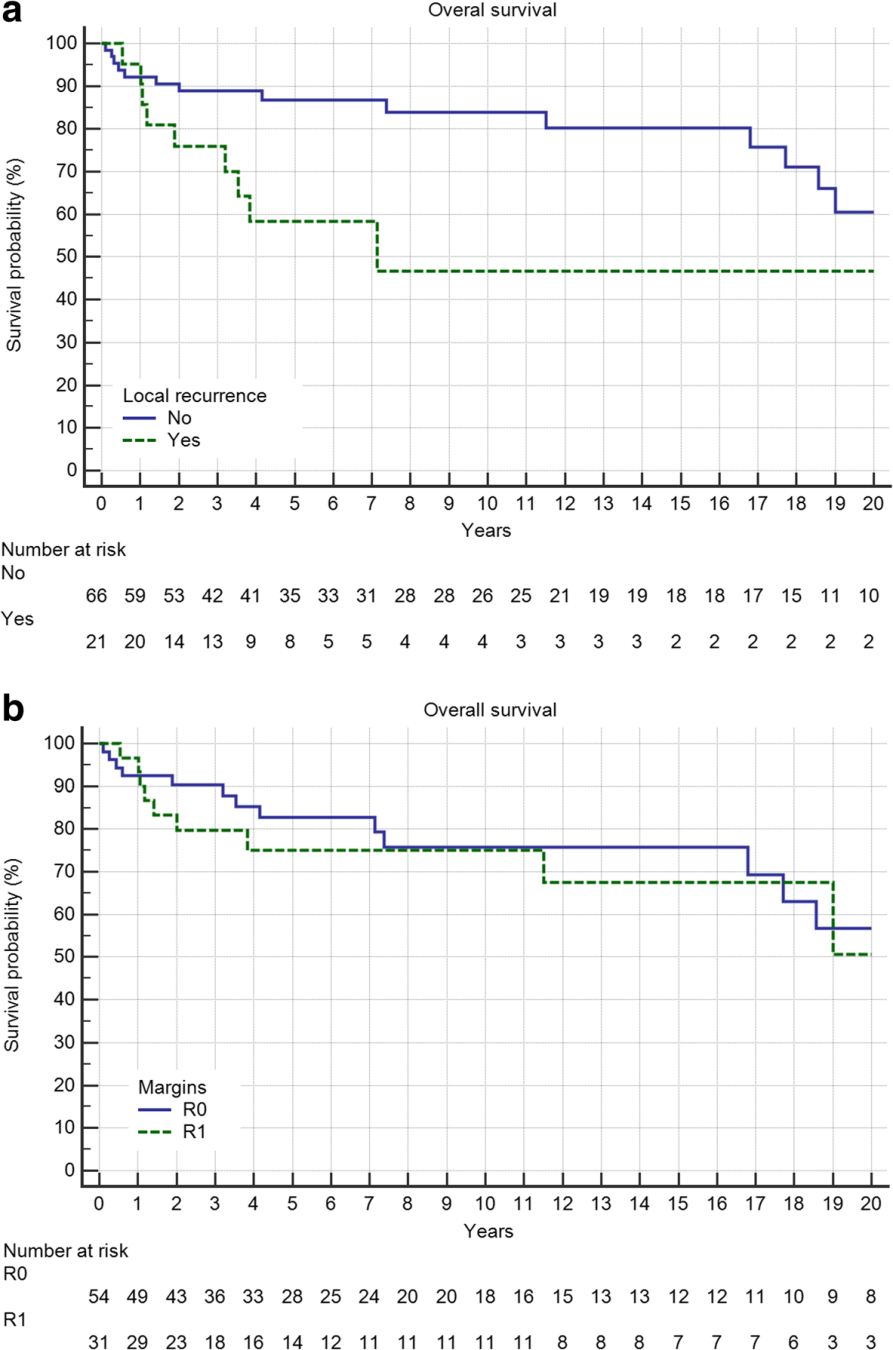
**a** Local recurrence in 21 patients reduces overall survival (*p* = 0.0219). **b** The surgical margin (R2 only 2 cases) does not influence overall survival (n.s)

**Fig. 7:**
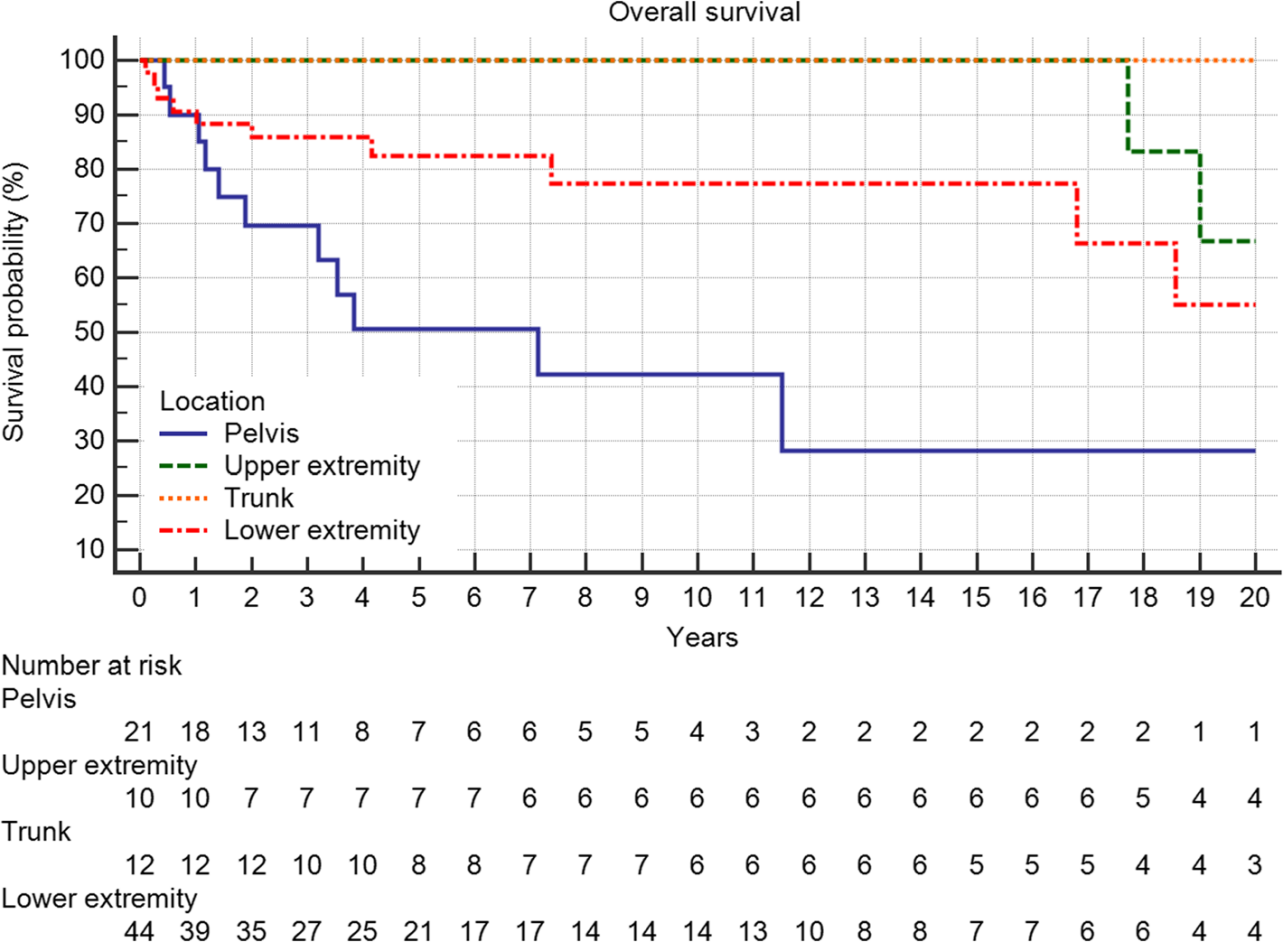
A pelvic location is worse in respect to overall survival (*p* = 0.0008)

Overall survival of the entire group at 5 and 10 years was 79 and 75%, respectively. Grading proved to be a significant factor (Fig. 4, *p* = 0.0099) as was metastatic disease (Fig. 5, *p* < 0.0001). Local recurrence also had a strong effect (Fig. 6a, *p* = 0.0219). Regarding margin status (Fig. 6b, n.s.) and localization (Fig. 7) only the latter had an influence on survival (*p* = 0.0008).

**Fig. 8:**
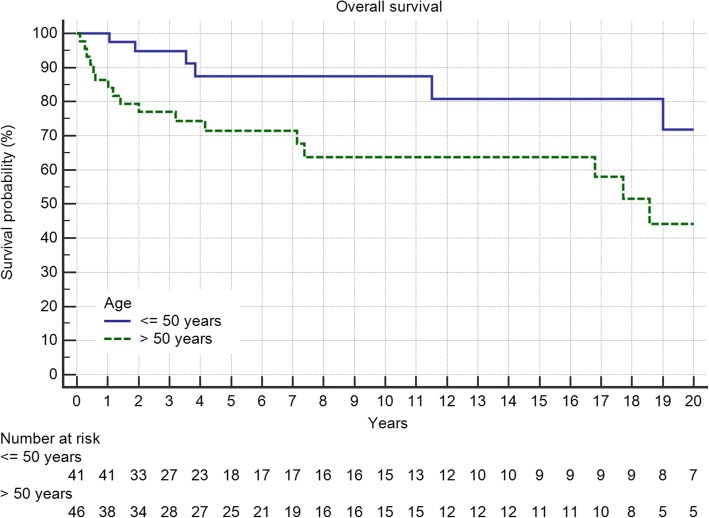
Overall survival is worse in patients older than 50 years (*p* = 0.019)

In general, male and female patients showed no difference whereas age with a cut-off of 50 years was a significant predictor of outcome (Fig. 8, *p* = 0.019).

As shown in Table 2, the quality of surgical margins and the presence of local recurrence did not influence overall survival in a multivariate analysis. Pelvic lesions led to a worse prognosis as did higher tumor grade, presence of metastatic disease and greater age.

**Table 2:** Cox proportional-hazards regression for overall survival in relation to grading, metastatic disease, age, margin status, location and local recurrence

Variables	Univariate analysis		Multivariate analysis	
	Hazard ratio (95% CI)	*p*-value	Hazard ratio (95% CI)	*p*-value
Grading	2532 (1360-4715)	**0,0034**	3208 (1380-7457)	**0,0067**
Metastatic disease	11,477 (4,6288-28,4589)	**< 0,0001**	14,763 (4819-45,229)	**< 0,0001**
Age < =50	2906 (1143-7389)	**0,0251**	0,307 (0,115-0,822)	**0,0188**
Margin status	1311 (0,642-2678)	0,4571	1152 (0,4997-2655)	0,7401
Pelvic/Non-pelvic	0,309 (0,165-0,578)	**0,0002**	0,441 (0,231-0,845)	**0,0136**
Local recurrence	2614 (1115-6125)	**0,0270**	1233 (0,448-3394)	0,6847

*P*-values in bold indicates significance

## Discussion

Age in general is a very strong factor of overall survival as shown in data out of the SEER Database (USA) [11]. Location is undoubtedly also an important aspect. As highly significantly shown in our data, pelvic location of a chondrosarcoma has a worse prognosis. This kept significance also in multivariate analysis. Our 5 and 10-year survival rates in those patients are 50 and 42%, respectively. In central chondrosarcomas, published 10-year survival rates vary between 54 and 88% [12–17]. This variability in survival depends very much on whether peripheral chondrosarcomas were included and how many patients in the study group had a low-grade chondrosarcoma or recurrent disease. Regarding margins, in pelvic lesions these were associated with LR [12, 13, 16, 17] but not OS [12, 15, 17]. In other studies LR did clearly influence OS [12–14]. However, the opposite observation, indications that LR did not influence OS has also been published [16]. Some authors showed that LR influenced metastatic disease and hence secondarily OS [13].

The main conclusion in summarizing the published literature and our own data is, that chondrosarcoma of the pelvis does exhibit a more aggressive behaviour and should not be curetted even in low-grade tumors. Local recurrence might lead to dedifferentiation and metastatic disease.

In general, low-grade central CS showed a good prognosis with a 5- and 10-year OS of 97 and 92%. But 5 of our 37 patients (14%) developed LR and 6 (16%) developed metastatic disease (MD), 4 of which eventually (11%) died from it. The published data on G1 chondrosarcoma is conflicting. From 0% LR and MD [18, 19], 2% LR and 0% MD [20], 3% LR and 3% MD [21], 4% LR and 0% MD [22], 5% LR and 0% MD [23], 6% LR and 0% MD [24, 25], 9% LR and 0% MD [26], 11% LR and 3% MD [27], 13% LR and 4% MD [28], 13% LR and 5% MD [29] to 18% LR and 6% MD [7] a variety of different results are reported. 5-year survival ranges from 82 to 99% and 10-year survival from 89 to 95% [8]. This reflects the problem of differentiation of benign enchondromas and atypical cartilaginous tumor and the heterogenous distribution of therapy and localization in these studies [3, 30–32]. Bauer et al. treated 40 patients with enchondromas and 40 patients with low-grade CS. His results showed no difference between groups [33]. So intralesional curettage with and without adjuvants is a valid option in most of those patients, but as stated above, central lesions should be resected because of their higher recurrence rates [27].

Metastatic disease was seen in 23%. This is about the same as described by other authors [10, 34–36]. There are series with a lesser [37, 38] or a higher [16, 39] proportion of metastatic disease. This reflects the importance of patient selection. The inclusion of initially non-metastasized patients only, patients with G2/3 lesions only or patients with axial or pelvic localizations only has a strong impact on MD and survival. In general MD is bad news for the patient with a 5-years overall survival of less than 50%. As shown in Table 2, MD is the most significant negative prognostic factor. There are patients, in whom metastatic disease is manageable by resection, local radiation or systemic therapy, leading to survival rates of 10–30% after 10 years, as also in this study. But this is the exception, mainly seen in G1 tumors [9, 34, 40]. Our results show, that MD is more common in G2/3 lesions as described by others [9, 34, 39, 41] but it is independent from surgical margins with the same rate of MD in R0 and R1 resected patients, and also independent from LR. This is in some respect in contrast to the literature [9, 10, 35] but other authors did see the same, confirming grade and location [42] as risk factors or grade as the only significant risk factor [16] for MD in multivariate analyses. In a large survey in Finland [36] the decade of diagnosis was the only significant factor on MD with an increased risk in the 1980s.

One of the most urgent questions is which margin should be obtained and how does margin influence LR and OS. In our study, local recurrence-free survival was significantly associated with margin status and LR influenced OS as in most of the published studies [9, 35, 43]. But in our data as well as in previous publications, LR and margin status showed no effect on overall survival in multivariate analysis [34]. We have to admit, that we only could include 2 cases with a R2 margin. Those seem to have a worse prognosis. There are not many studies including margin status in a multivariate analysis of overall survival [7, 10]. Lee shows a significant impact of margins on overall survival for patients with high-grade CS but the curves for wide and marginal resections did separate only after 120 months with just two events in the marginal group later on [10]. Fiorenza in 2002 reported findings identical to ours, namely a significant influence of LR on OS in univariate analysis and no influence of margin status in multivariate analysis [9]. LR remained significant as did grade and location. So in concordance with other groups, we conclude that LR after adequate resection is more likely to be a marker of the aggressiveness of the tumor than a consequence of failed local therapy [34, 44, 45]. We still maintain the premise of adequate resection, but some authors state that also intracompartmental grade 2 chondrosarcomas with a non-aggressive radiologic pattern can be treated by curettage without negatively affecting prognosis [46]. In patients with local recurrence but without MD, further aggressive surgery appears to constitute a good chance of cure (64% published by Fiorenza et al.) [9].

## Conclusions

The mainstay of therapy in chondrosarcoma of bone is surgery. Risk factors such as tumor grading, metastatic disease, age and location significantly influence overall survival. Margin status did influence local recurrence-free survival but not overall survival. Regarding the latter, the literature is inconclusive mainly due to a large heterogeneity of the study populations. Chondrosarcomas of the pelvis have a higher risk of local recurrence and should therefore be treated more aggressively at least to avoid local complications.

## Abbreviations

CS: Chondrosarcoma
CT: Computed Tomography
G1, G2, G3: Grading according to the French Federation of Cancer Centers grading system
LR: Local recurrence
LRFS: Local recurrence-free survival
MD: Metastatic disease
MRI: Magnetic resonance imaging
n.s.: Not significant
OS: Overall survival
R0, R1, R2: Resection margin
WHO: World Health Organization
